# Benign paroxysmal positional vertigo a systematic review of the effects of comorbidities

**DOI:** 10.3389/fneur.2025.1595693

**Published:** 2025-05-23

**Authors:** Haifa Alolayet, Louisa Murdin

**Affiliations:** ^1^Department of Audiology, Cochlear Implant Center, King Fahad Medical City, Riyadh, Saudi Arabia; ^2^Department of ENT, Guy’s and St Thomas NHS Foundation Trust and UCL Ear Institute, London, United Kingdom

**Keywords:** BPPV, occurrence, CRP, efficacy, comorbidity, risk factors

## Abstract

**Background:**

The prevalence of benign paroxysmal positional vertigo (BPPV) increases with age, as does the occurrence of other chronic health conditions. Although treatment with canalith-repositioning procedures (CRPs) is relatively successful, efficacy on the first attempt varies. Several studies have examined the influence of risk factors on BPPV occurrence and the efficacy of initial CRPs. However, findings are controversial. The objective of this study is to identify comorbidities associated with BPPV occurrence and explore their influence on the success of initial CRPs.

**Methods:**

The electronic databases PubMed, Scopus, Web of Science, Embase, MEDLINE and CINAHL were searched to identify eligible English original studies published from January 2019 to June 2024. All search results were reviewed based on our inclusion and exclusion criteria.

**Results:**

Of the 463 studies identified, 50 studies that satisfied the inclusion criteria were analysed. Eighteen studies focused on BPPV occurrence, 24 on the initial-CRP outcome and eight on both BPPV occurrence and the initial-CRP outcome. Twenty-five risk factors and comorbidities were identified to be associated with BPPV occurrence, and 15 were noted to impact the efficacy of the initial CRP. The most common reported risk factor for BPPV occurrence was head trauma (16 studies) and showed the poorest success rate after one CRP requiring a maximum of 18 manoeuvres to reach complete resolution (9 studies). Other factors included cardiovascular and endocrine comorbidities, neurological/neurotological comorbidities, musculoskeletal comorbidities, anxiety, obsessive-compulsive disorder, body mass index, serum vitamin D level and idiopathic BPPV.

**Conclusion:**

This systematic review assess the strength of evidence of risk factors influencing the development of BPPV and outcome of the initial CRP. Hypertension, hypotension, anaemia, ischaemic heart disease, hyperlipidaemia, stroke, diabetes mellitus, hypothyroidism, migraine, vestibular disorders, peripheral neuropathy, osteoporosis, cervical spondylosis, head trauma and low vitamin D were associated with BPPV incidence. Low levels of vitamin D, head trauma, migraine, inner ear diseases including Ménière’s disease, hypertension, high cholesterol, diabetes mellitus, hypothyroidism, hyperlipidaemia, osteoporosis, and reduced cervical mobility are all associated with failed first canal repositioning manoeuvre. There is an identified need to explore risk factors across different BPPV subtypes and their impact on the efficacy of various treatment manoeuvres.

## Introduction

Benign paroxysmal positional vertigo (BPPV) is one of the most common peripheral vestibular disorders, characterised by episodic vertigo provoked by head movement (1). BPPV arises through two distinct mechanisms: canalithiasis, the more widely accepted theory, where detached otoconia from the utricular macula float freely within the semicircular canal, or cupulolithiasis, where otoconia abnormally attach to the cupula (2).

The prevalence of BPPV increases with age, as does the occurrence of other acute or chronic health conditions (3–5). The majority of BPPV cases are idiopathic (6). Conversely, some individuals develop secondary BPPV from identifiable causes of otoconial displacement, which is seen in approximately 30% of cases (7).

BPPV can severely impact quality of life by reducing physical activity and increasing the risk of falls (6). It is treated with canalith-repositioning procedures (CRPs) to relocate the dislodged otoconia back into the vestibule (8). Although the treatment is relatively successful, its efficacy on the first attempt varies among patients (9). Evidence suggests that the underlying aetiology of BPPV may affect the treatment outcomes, delaying recovery (10). Several studies have examined the influence of risk factors and comorbidities on BPPV occurrence and the efficacy of initial CRPs. However, the findings are relatively controversial.

In this systematic review we aimed to determine the state of the science by examining the most recent evidence on the prevalence of comorbidities during the initial presentation of BPPV and their influence on the success of initial CRPs. Identifying these factors could significantly enhance clinical practice by facilitating the prevention of BPPV through mitigation of these risk factors, thereby reducing the impact of the condition on the health of high-risk populations. Moreover, understanding how comorbidities influence the effectiveness of CRPs enables clinicians to more accurately predict patient prognosis and anticipate the need for repeated CRP sessions. Additionally, identification of high-risk populations may optimise clinical outcomes, decrease the frequency of follow-up visits and improve the allocation of healthcare resources, ultimately enhancing the quality of care for patients experiencing BPPV.

## Methods

This study adhered to the Preferred Reporting Items for Systematic Reviews and Meta-analyses 2020 (PRISMA 2020) reporting guidelines to identify and assess studies (11).

### Literature search strategy

The electronic databases PubMed, Scopus, Web of Science, Embase, MEDLINE and CINAHL were systematically searched by two reviewers (HA and LM) for English studies published from January 2019 to June 2024. We developed a search strategy that included the following search terms: ‘occurrence’, ‘risk factor’, ‘comorbidity’, ‘BPPV’, ‘benign paroxysmal positional vertigo’, ‘CRP’, ‘CRM’, ‘canalith repositioning’, ‘efficacy’, ‘outcome’ and ‘success’. These terms were also combined with all related free words to search for relevant literature as comprehensively as possible. The detailed search strategy and process can be found in Supplementary Table 1.

### Selection process

All records identified were imported into an online tool (Covidence, 2024). Initially, reviewers screened the titles and abstracts of the identified studies using the eligibility criteria. Studies were included according to the following criteria: (1) adult patients over the age of 18 years diagnosed with any type of BPPV according to Bárány Society’s (12) criteria or equivalent guidelines (13); (2) BPPV treated with any type of CRP; (3) successful CRP defined as patients not showing positional vertigo or positional nystagmus; (4) sufficient data on comorbidities to answer either research question 1 or 2; (5) analysis of relevant comorbidities associated with BPPV occurrence; and (6) case–control, cohort or other observational studies. Articles were excluded according to the following criteria: (1) patients below the age of 18 years; (2) insufficient information on the diagnosis and treatment of BPPV; (3) BPPV recurrence as the study outcome; (4) insufficient information on the initial-CRP outcome; (5) non-peer-reviewed articles, non-original research, reviews, case studies/series/reports, letters, editorials or clinical protocols; and (6) animal studies. Following this, the remaining articles were retrieved for manual full-text review to identify any further relevant literature to establish the final set of studies ultimately included.

### Data extraction and quality assessment

The following data were extracted from the final set of included studies: author, publication year, study design, study location, sample size, mean participant age, comorbidities included, involved semicircular canal, outcome of the first CRP and number of CRPs conducted. The risk of bias for observational studies was assessed using the Newcastle–Ottawa Scale (NOS) (14, 15), and the randomised controlled trials (RCTs) were assessed using the Cochrane risk-of-bias tool for randomised trials (RoB 2) (16). The total NOS score is based on an accumulative score across three categories, a score of 7–9 is considered to indicate a low risk of bias; 4–6, an unclear risk of bias; and ≤3, a high risk of bias. RoB 2 tool assesses bias across five domains, studies are rated as *low* risk of bias if all domains indicate low bias; *unclear* risk of bias if at least one domain suggests high bias while others were low bias; and *high* risk of bias if at least one domains indicate high bias or multiple domains suggest unclear bias, potentially contributing to a loss of confidence in the results. The results of the NOS and RoB 2 quality assessment of the included studies are summarised in Tables 1, 2 and detailed description is given in Supplementary Tables 2–5.

**Table 1:** Characteristics of the included studies on the initial-CRP outcome.

Author and year	Sample size (case/control)	BPPV (n)	Design	Country	Mean patient age (SD), year	Basis of BPPV diagnosis	Risk factors and comorbidities included	Involved SCC	CRP	Outcome of the first CRP	CRP performed (n)	Quality score
Kim et al. (39)	63	63	Cohort	Korea	54 (n/a)	American Academy of Otolaryngology–Head and Neck Surgery guideline	Head trauma	Posterior SCC Horizontal SCC cupulolithiasis/canalolithiasis Anterior SCC Multicanal involvement	Epley manoeuvre Reversed Epley manoeuvre Barbecue roll manoeuvre Gufoni manoeuvre Barbecue roll manoeuvre with mastoid bone vibration	49% (31/63) failed	1 to 6 times in general	NOS: Low risk of bias
Jensen and Hougaard (46)	85	6	Cross-sectional	Denmark	43.46 ± 17.19	Bárány Society guideline	Head trauma	Posterior SCC canalolithiasis/cupulolithiasis Horizontal SCC canalolithiasis/cupulolithiasis Anterior SCC canalolithiasis Multicanal involvement	MRC Barbecue roll manoeuvre Deep head-hanging manoeuvre	All failed	Mean of 2 treatments per patient	NOS: Unclear risk of bias
McCormick and Kolar (36)	50	11	Cohort	USA	42.56 ± 17.72	American Academy of Otolaryngology–Head and Neck Surgery and Bárány Society guidelines	Head trauma	Posterior SCC canalolithiasis/cupulolithiasis Horizontal SCC canalolithiasis Bilateral posterior SCC canalithiasis	Appropriate CRP per BPPV based on the American Academy of Otolaryngology–Head and Neck Surgery guideline	8/11 failed	2 to 17 times for those who failed	NOS: Low risk of bias
Lou et al. (61)	230 (115/115)	230	RCT	China	Not reported	Bárány Society guideline	Idiopathic BPPV	Horizontal SCC canalolithiasis/cupulolithiasis Posterior SCC canalolithiasis	Manual and automatic swivel chair CRP: Barbecue manoeuvre Epley manoeuvre	65/230 failed	2 to 3 times for those who failed	RoB 2: High risk of bias
Lee et al. (62)	41	41	RCT	Korea	54.1 ± 10.4	No clear guidelines but BPPV definition similar to that in the Bárány Society/American Academy of Otorhinolaryngology–Head and Neck Surgery guideline	Idiopathic BPPV	Posterior SCC	Modified Epley manoeuvre with/without a pillow under the shoulders	7/41 failed	Not reported	RoB 2: Low risk of bias
Martellucci et al. (26)	69	69	Cohort	Italy	57.79 ± 15.05	No clear guidelines but BPPV definition similar to that in the Bárány Society/American Academy of Otorhinolaryngology–Head and Neck Surgery guideline	Idiopathic BPPV	Posterior SCC	Epley manoeuvre	30/69 failed	2 to 6 times for those who failed	NOS: Unclear risk of bias
Nahm et al. (24)	277	277	Cohort	Korea	Not reported	No clear guidelines but BPPV definition similar to that in the Bárány Society/American Academy of Otorhinolaryngology–Head and Neck Surgery guideline	Idiopathic BPPV	Posterior SCC Horizontal SCC canalolithiasis/cupulolithiasis Anterior SCC	Modified Epley manoeuvre Yacovino manoeuvre Barbecue roll manoeuvre Gufoni manoeuvre Appiani manoeuvre	133/277 failed	2 or more sessions	NOS: Low risk of bias
Fu et al. (25)	181	181	Cohort	China	51.98 ± 4.29 (for the 45–59-year age group) 70.17 ± 7.29 (for the over 60-year age group)	Bárány Society guideline	Idiopathic BPPV, HTN, DM	Posterior SCC Horizontal SCC	Epley manoeuvre Barbecue rotation manoeuvre Gufoni manoeuvre	51/181 failed	2 or 3 sessions	NOS: Low risk of bias
Lee et al. (63)	57	57	RCT	Korea	A: 50.8 ± 14.6 B: 50.7 ± 11.9	No clear guidelines but BPPV definition similar to that in the Bárány Society/American Academy of Otorhinolaryngology–Head and Neck Surgery guideline	Idiopathic BPPV	Horizontal SCC cupulolithiasis	A: Cupulolith-repositioning manoeuvre with mastoid oscillation B: New manoeuvre	A: 14/22 (63.6%) failed B: 25/35 (71.64%) failed	2 or 3 sessions	RoB 2: Unclear risk of bias
El-Anwar et al. (32)	114	114	Cohort	Egypt	43.2 ± 11.36	Bárány Society guideline	DM, HTN, hypothyroidism, head trauma, idiopathic BPPV	Posterior SCC Horizontal SCC Anterior SCC Bilateral SCC	Epley manoeuvre Barbecue manoeuvre Deep midline head-hanging manoeuvre All manoeuvres followed by post-procedure restriction for 1 week	16/114 (14%) failed	2 sessions	NOS: Low risk of bias
Wu et al. (23)	251	251	Cohort	China	55.2 ± 14.3	American Academy of Otolaryngology–Head and Neck Surgery and Bárány Society guidelines	Serum vitamin D level, osteoporosis, vascular comorbidities (e.g., HTN, DM, coronary heart disease and hyperlipidaemia)	Posterior SCC Horizontal SCC	Epley manoeuvre Barbecue manoeuvre Gufoni manoeuvre	89/251 failed	Not reported	NOS: Low risk of bias
Khaftari et al. (64)	43	43	RCT	Iran	52.18 ± 8.16	American Academy of Otolaryngology–Head and Neck Surgery guideline	Idiopathic BPPV	Posterior SCC	Half Somersault manoeuvre Epley manoeuvre	39% of the Epley manoeuvre group failed 65% of the half Somersault manoeuvre group failed	2 to 4 sessions	RoB 2: Unclear risk of bias
Kjærsgaard et al. (65)	70	70	RCT	Denmark	A: 60.9 ± 16.8 B: 63.7 ± 14.6	American Academy of Otolaryngology–Head and Neck Surgery and Bárány Society guidelines	Idiopathic BPPV	Posterior SCC canalolithiasis	A: Conventional Epley manoeuvre with the MRC B: Potentiated version of the Epley manoeuvre with the MRC	A: 18/33 (54.5%) failed B: 10/28 (35.7%) failed	2 to 10 sessions	RoB 2: Unclear risk of bias
Lee et al. (66)	96	96	Cohort	Taiwan	Epley: 55.9 ± 15.3 Epley + supine to prolonged lateral position: 52.5 ± 12	No clear guidelines but BPPV definition similar to that in the Bárány Society/American Academy of Otorhinolaryngology–Head and Neck Surgery guideline	HTN, hyperlipidaemia, idiopathic BPPV	Posterior SCC	Epley manoeuvre Epley manoeuvre + supine to prolonged lateral position	32/96 failed	2 or more sessions	NOS: Low risk of bias
Yang et al. (31)	40	40	Cohort	Korea	61.43 ± 13.14	Bárány Society guideline	HTN, DM, hyperlipaemia, coronary heart disease, cerebral infarction, SNHL, TBI	Anterior SCC cupulolithiasis/canalolithiasis Multicanal involvement	Yacovino manoeuvre	23/40 failed	Not reported	NOS: Low risk of bias
Zhang et al. (30)	36	36	Cohort	China	49.1 ± 14.9	No clear guidelines but BPPV definition similar to that in the Bárány Society/American Academy of Otorhinolaryngology–Head and Neck Surgery guideline	Head trauma	Posterior SCC Horizontal SCC Multicanal involvement	Computer-controlled repositioning Computer-controlled Epley manoeuvre Computer-controlled barbecue manoeuvre Computer-controlled Gufoni manoeuvre	22/36 (61.1%) failed	2 to 18 sessions	NOS: Low risk of bias
Gupta and Solanki (29)	120	120	Cohort	India	43.5 (n/a)	No clear guidelines but BPPV definition similar to that in the Bárány Society/American Academy of Otorhinolaryngology–Head and Neck Surgery guideline	Head trauma, HTN, DM, hypothyroidism, migraine, hyperlipidaemia	Posterior SCC	Epley manoeuvre	12/120 (10%) failed	2 to 4 sessions	NOS: Low risk of bias
Chen et al. (60)	65	65	RCT	China	Control: 51.59 ± 14.74 Experimental: 7.09 ± 14.3	Bárány Society guideline	HTN, DM, idiopathic BPPV	Posterior SCC	Epley manoeuvre Modified Epley manoeuvre	17/65 failed	2 to 3 sessions	RoB 2: Unclear risk of bias
Imai et al. (59)	180	180	RCT	Japan	A: 70.7 (n/a) B: 71.1 (n/a)	American Academy of Otolaryngology–Head and Neck Surgery and Bárány Society guidelines	Idiopathic BPPV	Posterior SCC	A: Epley manoeuvre B: Repeated Dix–Hallpike manoeuvre	28/180 failed	2 to 3 sessions	RoB 2: Unclear risk of bias
Maas et al. (21)	102	102	Cohort	Netherlands	Median reported	Bárány Society guideline	Idiopathic BPPV	Horizontal SCC	Lempert manoeuvre Gufoni manoeuvre Modified Gufoni manoeuvre Head-shaking manoeuvre	33/102 failed	2 or more sessions	NOS: Low risk of bias
Nadagoud et al. (58)	90	90	RCT	India	45.34 ± 10.96	No clear guidelines but BPPV definition similar to that in the Bárány Society/American Academy of Otorhinolaryngology–Head and Neck Surgery guideline	Head trauma, HTN, DM, idiopathic BPPV	Posterior SCC	Epley manoeuvre Semont manoeuvre Gans manoeuvre	15/90 failed	2 or 3 sessions	RoB 2: Unclear risk of bias
Han et al. (57)	54	54	RCT	Korea	Not reported	American Academy of Otolaryngology–Head and Neck surgery guideline	Idiopathic BPPV	Horizontal SCC canalolithiasis	Cupulolith-repositioning manoeuvre	46/54 (85.2%) failed	2 to 4 sessions	RoB 2: Unclear risk of bias
Celis-Aguilar et al. (56)	38	38	RCT	Mexico	59.85 ± 13.10	No clear guidelines but BPPV definition similar to that in the Bárány Society/American Academy of Otorhinolaryngology–Head and Neck Surgery guideline	DM, HTN, hypothyroidism, dyslipidaemia, idiopathic BPPV	Posterior SCC	Brandt–Daroff exercises Semont manoeuvre Epley manoeuvre	18/34 failed (4/38 lost to follow-up)	2 sessions	RoB 2: Unclear risk of bias
Saruhan et al. (53)	80 (40/40)	40	Case–control	Türkiye	52.3 ± 9.0	No clear guidelines but BPPV definition similar to that in the Bárány Society/American Academy of Otorhinolaryngology–Head and Neck Surgery guideline	Idiopathic BPPV	Posterior SCC	Modified Epley manoeuvre	6/40 failed	2 sessions	NOS: Low risk of bias
Kher (42)	200	200	Cross-sectional	India	44.50 ± 12.20	Bárány Society guideline	HTN, DM, hypothyroidism, head trauma, idiopathic BPPV	Posterior SCC Horizontal SCC Anterior SCC	Epley manoeuvre Barbecue roll manoeuvre Deep midline head-hanging manoeuvre	28/200 (14%) failed	2 sessions	NOS: Unclear risk of bias
Kong et al. (55)	49	49	RCT	Korea	57.29 ± 14.97	American Academy of Otolaryngology–Head and Neck Surgery guideline	Idiopathic BPPV	Horizontal SCC	Modified Lempert manoeuvre Head-shaking manoeuvre Cupulolith-repositioning manoeuvre	35/49 failed	2 sessions	RoB 2: High risk of bias
De Hertogh et al. (20)	148	88	Cohort	Belgium	59.3 ± 16.22	American Academy of Otolaryngology–Head and Neck Surgery guideline	DM, neck pain, idiopathic BPPV	Posterior SCC Horizontal SCC Bilateral SCC	Epley manoeuvre Lempert manoeuvre	47/83 failed (5/88 spontaneous recovery)	2 to 8 sessions	NOS: Unclear risk of bias
Lee J et al. (22)	25	25	RCT	Korea	A: 53.3 ± 11.9 B: 50.3 ± 14.1	No clear guidelines but BPPV definition similar to that in the Bárány Society/American Academy of Otorhinolaryngology–Head and Neck Surgery guideline	Idiopathic BPPV	Horizontal SCC	A: Gufoni manoeuvre B: Appiani manoeuvre	21/25 failed	2 to 3 sessions	RoB 2: Unclear risk of bias
Schuricht and Hougaard (54)	74	74	RCT	Denmark	TRV: 56.6 ± 17.6 Manual treatment: 63.9 ± 14.3	American Academy of Otorhinolaryngology–Head and Neck Surgery guideline	Idiopathic BPPV	Posterior SCC canalolithiasis/cupulolithiasis Horizontal SCC canalolithiasis/cupulolithiasis Multicanal involvement	Epley manoeuvre Lempert manoeuvre TRV chair	28/74 failed	2 to 14 sessions	RoB 2: Unclear risk of bias
Zhao et al. (19)	254	254	Cohort	China	53.52 ± 13.86	No clear guidelines but BPPV definition similar to that in the Bárány Society/American Academy of Otorhinolaryngology–Head and Neck Surgery guideline	Idiopathic BPPV	Horizontal SCC	Gufoni manoeuvre Li manoeuvre	98/254 failed	2 to 3 sessions	NOS: Low risk of bias
Martellucci et al. (18)	47	47	Cohort	Italy	62.06 ± 13.09	Bárány Society guideline	Reduced cervical mobility	Posterior SCC	Epley manoeuvre	18/47 (38.3%) failed	2 to 4 sessions	NOS: Low risk of bias
Jiang et al. (17)	220	220	Cohort	China	57.2 (n/a)	American Academy of Otorhinolaryngology–Head and Neck Surgery and Bárány Society guidelines	Idiopathic BPPV	Posterior SCC Horizontal SCC	Epley manoeuvre Barbecue manoeuvre	83/220 failed	2 or 3 sessions	NOS: Low risk of bias

A quality score in (green) indicates a low risk of bias; (yellow) indicates an unclear risk of bias; (red) indicates a high risk of bias. BPPV, benign paroxysmal positional vertigo; SCC, semicircular canal; CRP, canalith-repositioning procedure; n/a, not available; TRV, Thomas Richard Vitton; MRC, mechanical rotational chair; NOS, Newcastle–Ottawa Scale; RoB 2, risk-of-bias tool for randomised trials; RCT, randomised controlled trial; DM, diabetes mellitus; HTN, hypertension; TBI, traumatic brain injury; SNHL, sensorineural hearing loss.

**Table 2:** Pooled results of the participant characteristics associated with the first BPPV occurrence.

Comorbidity	Number of included studies	Number of participants	Number of studies reporting an association with BPPV occurrence	Quality of studies reporting an association with BPPV occurrence
Cardiovascular comorbidities				
Hypertension	14	5,805	10	9 Good-quality, 1 fair-quality
Hypotension	2	769	1	Good-quality
Anaemia	1	320	1	Good-quality
Ischaemic/coronary heart disease	4	588	2	Good-quality
Hyperlipidaemia/dyslipidaemia	11	5,431	7	Good-quality
Stroke	4	5,882	3	Good-quality
Endocrine comorbidities				
Diabetes mellitus	14	5,805	10	9 Good-quality, 1 fair-quality
Hypothyroidism	4	489	4	3 Good-quality, 1 fair-quality
Thyroid disease	2	791	0	n/a
Psychiatric comorbidities				
Depression	1	618	0	n/a
Anxiety/obsessive-compulsive disorder	1	2,612	1	Good-quality
Neurological comorbidities				
Migraine	6	1,211	6	5 Good-quality, 1 fair-quality
Labyrinthitis	2	699	2	Good-quality
Neuritis	1	457	1	Good-quality
Meniere’s disease	3	1,156	3	Good-quality
SNHL	3	273	2	Good-quality
Sleep disorder (insomnia)	1	2,612	1	Good-quality
Peripheral neuropathy	1	618	1	Good-quality
Musculoskeletal comorbidities				
Osteopenia	2	87	2	Good-quality
Osteoporosis	7	4,240	6	Good-quality
Cervical spondylosis	1	2,612	1	Good-quality
Other conditions/factors				
Head trauma	16	2,169	16	11 Good-quality, 5 fair-quality
BMI	3	816	1	Good-quality
Serum vitamin D level	8	1,123	8	7 Good-quality, 1 fair-quality
Kidney disease	1	173	0	n/a

BPPV, benign paroxysmal positional vertigo; CRP, canalith-repositioning procedure; SNHL, sensorineural hearing loss.

## Results

### Study characteristics

**Figure 1:**
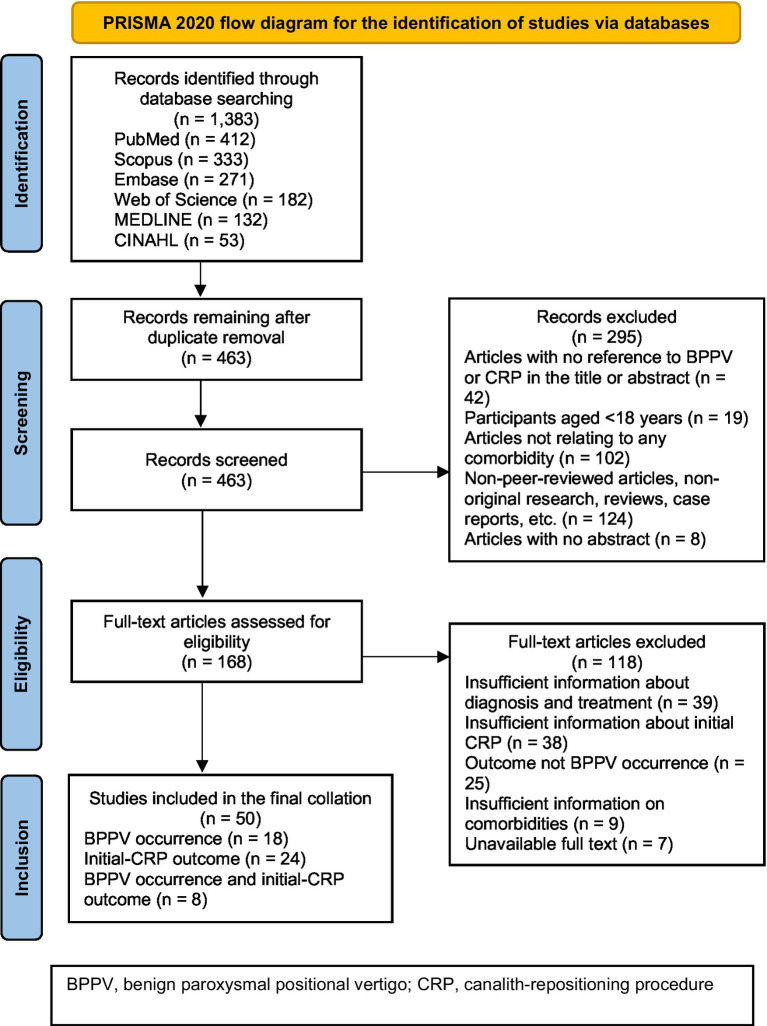
Flow diagram detailing the search and study selection processes.

A total of 1,383 studies were initially identified for screening. The review ultimately included 50 studies (17–66) 18 studies on BPPV occurrence, 24 studies on the initial-CRP outcome and eight studies on both BPPV occurrence and the initial-CRP outcome. Reasons for exclusion are described in Figure.1.

Of the 50 studies, twenty-five studies were cohort studies; six were cross-sectional studies; six were case–control studies; and 13 were randomised control trials. Half of the studies (*n* = 25) had fewer than 100 participants, and the research included studies from 18 different countries, with no geographical restrictions. BPPV diagnosis was based on the Bárány Society guideline (12) or American Academy of Otolaryngology–Head and Neck Surgery guideline (13) for BPPV in most studies (*n* = 33); the remaining studies (*n* = 17) did not specify which guideline they used but reported their diagnostic methods and their findings. In regard to quality assessment, 30 studies were considered to have a low risk of bias; 18, an unclear risk of bias; and two, a high risk of bias (Tables 1, 3).

**Table 3:** Characteristics of the included studies on BPPV occurrence.

Author and year	Sample size (case/control)	Design	Country	Mean patient age (SD), year	Basis of BPPV diagnosis	Risk factors and comorbidities included	Findings	Quality score
Bi et al. (49)	52 (27/25)	Case–control	China	51.63 ± 15.82	American Academy of Otolaryngology–Head and Neck Surgery guideline	Osteopenia, osteoporosis, serum vitamin D level	The association with BPPV occurrence was statistically examined.	NOS: Low risk of bias
Ding et al. (48)	522 (174/348)	Cross-sectional	China	Median reported	American Academy of Otolaryngology–Head and Neck Surgery guideline	Serum vitamin D level, DM, HTN, hyperlipidaemia	DM, HTN and hyperlipidaemia were background health characteristics when patients were first diagnosed with BPPV. Only the serum vitamin D level was examined.	NOS: Low risk of bias
Cheng et al. (50)	640 (320/320)	Case–control	China	68.2 ± 6.02	American Academy of Otolaryngology–Head and Neck Surgery guideline	DM, HTN, osteoporosis, anaemia, hyperlipidaemia, coronary heart disease, serum vitamin D level, BMI	The association with BPPV occurrence was statistically examined.	NOS: Low risk of bias
Ren et al. (51)	364 (182/182)	Case–control	China	Median reported	American Academy of Otolaryngology–Head and Neck Surgery guideline	Serum vitamin D level, BMI	The association with BPPV occurrence was statistically examined.	NOS: Low risk of bias
Ghosh and Dorasala (27)	151	Cohort	India	Not reported	No clear guidelines but BPPV definition similar to that in the Bárány Society/American Academy of Otorhinolaryngology–Head and Neck Surgery guideline	Vestibular migraine, Meniere’s disease, labyrinthitis, orthostatic hypotension	The association with BPPV occurrence was statistically examined.	NOS: Low risk of bias
Kim et al. (41)	255	Cohort	USA	63.0 ± 13.4	Bárány Society guideline	Migraine	The association with BPPV occurrence was statistically examined.	NOS: Unclear risk of bias
Cobb et al. (47)	6,135 (173/5,962)	Cross-sectional	USA	66.2 ± 11.8	No clear guidelines but BPPV definition similar to that in the Bárány Society/American Academy of Otorhinolaryngology–Head and Neck Surgery guideline	Migraine, serum vitamin D level, HTN, DM, hyperlipidaemia, kidney disease, heart disease, thyroid disease, hearing loss	The association with BPPV occurrence was statistically examined.	NOS: Low risk of bias
Andersson et al. (40)	117	Cohort	Norway	50 (n/a)	Bárány Society guideline	Head trauma	The association with BPPV occurrence was statistically examined.	NOS: Low risk of bias
Kim et al. (39)	63	Cohort	Korea	54 (n/a)	American Academy of Otolaryngology–Head and Neck Surgery guideline	Head trauma	The association with BPPV occurrence was statistically examined.	NOS: Low risk of bias
Jensen and Hougaard (46)	85	Cross-sectional	Denmark	43.46 ± 17.19	Bárány Society guideline	Head trauma	The association with BPPV occurrence was statistically examined.	NOS: Unclear risk of bias
Jafarzadeh et al. (45)	21	Cross-sectional	India	45.0 ± 12.7	No clear guidelines but BPPV definition similar to that in the Bárány Society/American Academy of Otorhinolaryngology–Head and Neck Surgery guideline	Head trauma	The association with BPPV occurrence was statistically examined.	NOS: Unclear risk of bias
Álvarez-Morujo de Sande et al. (38)	457	Cohort	Spain	Median reported	Bárány Society guideline	Migraine, TBI, head trauma, Meniere’s disease, vestibular neuritis, hyperlipidaemia, HTN, DM	The association with BPPV occurrence was statistically examined.	NOS: Low risk of bias
Elmoursy and Abbas (37)	60	Cohort	Egypt	46.02 ± 12.56	American Academy of Otolaryngology–Head and Neck Surgery guideline	Serum vitamin D level, HTN, DM, head trauma, osteopenia, osteoporosis, SNHL	HTN, DM, head trauma, osteopenia, osteoporosis and SNHL were background health characteristics when patients were first diagnosed with BPPV. Only the serum vitamin D level was examined.	NOS: Low risk of bias
McCormick and Kolar (36)	50	Cohort	USA	42.56 ± 17.72	American Academy of Otolaryngology–Head and Neck Surgery and Bárány Society guidelines	Head trauma	The association with BPPV occurrence was statistically examined.	NOS: Low risk of bias
Harrell et al. (44)	73	Cross-sectional	USA	53.9 ± 22.4	No clear guidelines but BPPV definition similar to that in the Bárány Society/American Academy of Otorhinolaryngology–Head and Neck Surgery guideline	Head trauma	The association with BPPV occurrence was statistically examined.	NOS: Unclear risk of bias
Yehuda et al. (35)	55	Cohort	Israel	Median reported	American Academy of Otolaryngology–Head and Neck Surgery guideline	HTN, DM, high cholesterol level, osteoporosis, vitamin D deficiency, head injury, heart or blood vessel problems, hypothyroidism, migraine	All were background health characteristics when patients were first diagnosed with BPPV.	NOS: Low risk of bias
Shu et al. (34)	2,612	Cohort	China	52.98 (n/a)	Bárány Society guideline	Dyslipidaemia, osteoporosis, cervical spondylosis, posterior-circulation ischaemia, cerebral infarction, DM, HTN, obsessive-compulsive disorder, insomnia, anxiety	All were background health characteristics when patients were first diagnosed with BPPV.	NOS: Low risk of bias
Singh et al. (52)	628 (314/314)	Case–control	USA	Not reported	No clear guidelines but BPPV definition similar to that in the Bárány Society/American Academy of Otorhinolaryngology–Head and Neck Surgery guideline	HTN, DM, BMI, dyslipidaemia	The association with BPPV occurrence was statistically examined.	NOS: Low risk of bias
Hyland et al. (43)	618	Cross-sectional	Australia	77.7 ± 9.3	American Academy of Otolaryngology–Head and Neck Surgery guideline	Osteoporosis, DM, thyroid disease, HTN, stroke, hyperlipidaemia, depression, postural hypotension, peripheral neuropathy	The association with BPPV occurrence was statistically examined.	NOS: Low risk of bias
Kong et al. (33)	548	Cohort	Korea	54.3 ± 14.6	American Academy of Otorhinolaryngology–Head and Neck Surgery guideline	Head trauma, labyrinthitis, Meniere’s disease, HTN, DM, hyperlipidaemia, osteoporosis	All were background health characteristics when patients were first diagnosed with BPPV.	NOS: Low risk of bias
El-Anwar et al. (32)	114	Cohort	Egypt	43.2 ± 11.36	Bárány Society guideline	DM, HTN, hypothyroidism, head trauma	All were background health characteristics when patients were first diagnosed with BPPV.	NOS: Low risk of bias
Yang et al. (31)	40	Cohort	Korea	61.43 ± 13.14	Bárány Society guideline	HTN, DM, hyperlipaemia, coronary heart disease, cerebral infarction, deafness, TBI	All were background health characteristics when patients were first diagnosed with BPPV.	NOS: Low risk of bias
Zhang et al. (30)	36	Cohort	China	49.1 ± 14.9	No clear guidelines but BPPV definition similar to that in the Bárány Society/American Academy of Otorhinolaryngology–Head and Neck Surgery guideline	Head trauma	The association with BPPV occurrence was statistically examined.	Low risk of bias
Gupta and Solanki (29)	120	Cohort	India	43.5 (n/a)	No clear guidelines but BPPV definition similar to that in the Bárány Society/American Academy of Otorhinolaryngology–Head and Neck Surgery guideline	Head trauma, HTN, DM, hypothyroidism, migraine, hyperlipidaemia	All were background health characteristics when patients were first diagnosed with BPPV.	NOS: Low risk of bias
Martens et al. (28)	132	Cohort	Norway	57 ± 13	Bárány Society guideline	Head trauma, serum vitamin D level	Head trauma was a background health characteristic when patients were first diagnosed with BPPV. Only the serum vitamin D level was examined.	NOS: Unclear risk of bias
Kher (42)	200	Cross-sectional	India	44.50 ± 12.20	Bárány Society guideline	HTN, DM, hypothyroidism, head trauma	All were background health characteristics when patients were first diagnosed with BPPV.	NOS: Unclear risk of bias

A quality score in (green) indicates a low risk of bias; (yellow) indicates an unclear risk of bias. BPPV, benign paroxysmal positional vertigo; n/a, not available; NOS, Newcastle–Ottawa Scale; DM, diabetes mellitus; HTN, hypertension; BMI, body mass index; TBI, traumatic brain injury; SNHL, sensorineural hearing loss.

Among the 26 included studies on BPPV occurrence (Table 3), 28 risk factors and comorbidities were identified. Seven studies presented the risk factors and comorbidities as background health characteristics when patients were first diagnosed with BPPV and were not statistically studied. Three studies examined only the serum vitamin D level and presented the remaining risk factors as the baseline characteristics of BPPV occurrence. The remaining 16 studies examined the statistical association between the risk factors and BPPV occurrence.

Of the 32 included studies on the efficacy of the first CRP (Table 1), 15 risk factors and comorbidities were identified. The most commonly involved semicircular canal was the posterior canal (*n* = 25), followed by the horizontal canal (*n* = 18), the anterior canal (n = 6), the bilateral canals (*n* = 3) and multiple canals (*n* = 5) (Table 1). Across all studies, 24 different CRP manoeuvres were employed. Three studies did not report the number of manoeuvres performed after participants failed the first CRP.

#### Synthesised findings

##### Risk factors and comorbidities associated with BPPV occurrence

Twenty-eight risk factors and comorbidities were identified (Table 2). The most common risk factor for BPPV occurrence was head trauma, reported in eleven good-quality studies and five fair-quality studies involving a total of 2,169 patients. Cardiovascular comorbidities were explored in 36 studies, with hypertension reported in 14 studies involving 5,805 patients; only 10 (28%) showed an association with BPPV occurrence, primarily from good-quality studies. Hyperlipidaemia and dyslipidaemia were identified as risk factors in seven out of 11 (64%) good-quality studies with 5,431 participants. Stroke was investigated in four studies involving 5,882 participants, and three studies of good-quality showed an association with BPPV. Out of the four good-quality studies on ischaemic and coronary heart diseases involving 588 participants, only two studies reported a link with BPPV occurrence. Two good-quality studies involving 769 participants investigated hypotension as a factor, and the association with BPPV incidence was reported in one study. A single study of good-quality involving 320 participants reported an association between anaemia and BPPV occurrence.

Eight studies involving 1,123 patients reported the serum vitamin D level as a risk factor for the first occurrence of BPPV, this association was supported primarily from good-quality studies. Among the three good-quality studies on body mass index (BMI) involving 816 participants, only one study reported BMI as a risk factor for BPPV occurrence. A single good-quality study involving 173 participants investigated kidney disease and showed no association with BPPV occurrence. Twenty studies analysed the influence of endocrine comorbidities on BPPV occurrence. Ten out of the fourteen studies involving 5,805 participants reported diabetes mellitus as a risk factor, with most findings coming good-quality studies. An association between BPPV occurrence and hypothyroidism was reported in four studies involving 489 participants. Three of the studies were of good-quality, while one had fair-quality. Two good-quality studies involving 791 participants investigated the association between thyroid disease and the first presentation of BPPV, and none of them showed any association.

Migraine, labyrinthitis, neuritis, Meniere’s disease, sensorineural hearing loss (SNHL), sleep disorder (insomnia) and peripheral neuropathy were reported in 17 studies. Six studies involving 1,211 participants indicated migraine as a risk factor for BPPV occurrence. This association was based on findings coming primarily from good-quality studies. The correlation between Meniere’s disease and BPPV occurrence was investigated in three studies of good-quality involving 1,156 patients with BPPV. Two of the three good-quality studies including 273 patients with BPPV showed the impact of SNHL on BPPV occurrence. Labyrinthitis was reported as a risk factor in two good-quality studies involving 273 participants. A single good-quality study involving 457 participants found neuritis as a risk factor for BPPV occurrence. Two studies of good-quality involving 3,230 patients analysed the influence of sleep disorder (insomnia) and peripheral neuropathy on BPPV occurrence.

Psychiatric comorbidities were investigated in two good-quality studies involving 3,230 participants. Anxiety and obsessive-compulsive disorder were found to be associated with BPPV occurrence, while depression was not. Studies of good-quality analysed the influence of musculoskeletal comorbidities on BPPV occurrence. Two studies including 87 and 2,612 participants reported osteopenia and cervical spondylosis, respectively, as a risk factor for BPPV occurrence. Six out of the seven studies involving 4,240 patients with BPPV reported the influence of osteoporosis on BPPV development.

##### Risk factors and comorbidities affecting the efficacy of initial CRPs

Fifteen risk factors and comorbidities were identified to affect the efficacy of initial CRPs (Table 4). Head trauma was reported in nine studies involving 680 participants where up to 18 manoeuvres were needed to reach complete resolution. This association was based on findings from six good-quality and three fair-quality studies. Idiopathic BPPV was assessed in 24 studies involving 2,594 participants, requiring 14 manoeuvres for complete symptom resolution. Nine of the studies were of good-quality, thirteen had fair-quality and two were of poor-quality. Two good-quality studies involving 331 participants showed that the serum vitamin D level, osteoporosis, SNHL and stroke (cerebral infarction) were risk factors for a poorer response to the first CRP; however, the number of manoeuvres performed to reach complete resolution was not reported in these studies.

**Table 4:** Pooled results of the participant characteristics associated with failed first CRP.

Comorbidity	Number of studies	Number of participants	Maximum number of reported CRP	Quality of studies reporting poor efficacy of initial CRPs
Cardiovascular comorbidities				
Hypertension	10	1,195	4 sessions	6 Good-quality, 4 fair-quality
Coronary heart disease	2	291	Not reported	Good-quality
Hyperlipidaemia/dyslipidaemia	5	545	4 sessions	4 Good-quality, 1 fair-quality
Stroke (cerebral infarction)	1	40	Not reported	Good-quality
Endocrine comorbidities				
Diabetes mellitus	10	1,187	8 sessions	5 Good-quality, 5 fair-quality
Hypothyroidism	4	472	4 sessions	2 Good-quality, 2 fair-quality
Neurological comorbidities				
Migraine	1	120	4 sessions	Good-quality
SNHL	1	40	Not reported	Good-quality
Musculoskeletal comorbidities				
Osteoporosis	1	251	Not reported	Good-quality
Reduced cervical mobility/neck pain	2	135	8 sessions	1 Good-quality, 1 fair-quality
Other conditions/factors				
Head trauma	9	680	18 sessions	6 Good-quality, 3 fair-quality
Serum vitamin D level	1	251	Not reported	Good-quality
Idiopathic BPPV	24	2,594	14 sessions	9 Good-quality, 13 fair-quality, 2 poor-quality

BPPV, benign paroxysmal positional vertigo; CRP, canalith-repositioning procedure; SNHL, sensorineural hearing loss.

The impact of coronary heart disease was shown in two studies of good-quality involving 291 participants, with no reported data on the number of manoeuvres needed for successful treatment. Endocrine comorbidities including hypothyroidism and diabetes mellitus reported in 14 studies involving 1,659 participants reduced the efficacy of the first CRP, and participants needed a maximum of four to eight sessions, respectively, for complete recovery. This association was based on findings from seven good-quality studies and seven fair-quality studies with an unclear risk. The effect of reduced cervical mobility and neck pain on treatment efficacy after one manoeuvre was demonstrated in two studies of fair-to-good quality including 135 participants; these risk factors increased the probability of an increased number of CRPs performed, and data showed that a maximum of eight sessions were needed to reach complete resolution.

The influence of migraine on the efficacy of the first treatment was reported in a single good-quality study involving 120 participants, and the maximum number of manoeuvres necessary to achieve complete symptom resolution was four. Ten studies including 1,195 patients with BPPV showed that participants with hypertension needed a maximum of four manoeuvres to achieve the resolution of nystagmus and remission of BPPV symptoms. Six of the studies were of good-quality, while four had fair-quality. Five studies involving 545 participants indicated hyperlipidaemia and dyslipidaemia as risk factors that worsen the prognosis of the first CRP, and the maximum number of manoeuvres needed to reach complete resolution of symptoms was four. Four of these studies were of good-quality and one was of fair-quality.

## Discussion

Our systematic review of 50 studies summarised the recent literature from the last 5 years on the risk factors and comorbid condition associated with the occurrence of BPPV, as well as their influence as outcome predictors of the success of initial CRP treatment. Twenty-five risk factors were identified as associated with BPPV development, while 15 were associated with poorer outcome from the first CRP. Our study builds on previous work, reviewing the most recent studies, and, in contrast to some previous reviews, using a robust definition of BPPV. Furthermore, our work, unlike previous reviews, has included a formal evaluation of study methodology using a risk of bias tool to allow appropriate weighting of the different included studies.

### BPPV occurrence

#### Bone mineral density and serum vitamin D level

Our findings suggest that low levels of vitamin D are a risk factor for BPPV occurrence. This is consistent with recent meta-analysis (67). Vitamin D plays a major role in calcium metabolism, which can affect the density and matrix of otoconia, potentially leading to BPPV (68). Moreover, studies suggest that the severity of BPPV attacks and their recurrence are correlated with vitamin D insufficiency (69, 70). Previous studies have found lower bone mineral density in BPPV patients compared to controls (71, 72). Our pooled results indicated that osteoporosis and osteopenia are risk factors for the occurrence of BPPV. This result was consistent with a previous systematic review (73). Osteoporosis is reported to be associated with a 1.34 times higher odds of developing BPPV (74). Moreover, the treatment of vitamin D deficiency and osteoporosis is reported in some studies to have a protective effect against BPPV occurrence and improve recovery by reducing relapses of attacks and BPPV recurrence (70, 75).

#### Head trauma and migraine

Consistent with previous studies (76, 77), this review presents evidence suggesting that BPPV is strongly linked to head trauma or migraine. Pisani et al. (78) in an observational study of 3,060 BPPV patients, found a clear association with traumatic events in 23.4% of cases. A national epidemiological survey also reported interesting findings, highlighting that the prevalence of migraine in BPPV patients was twice as high as that in age-and sex-matched controls (6). The pathophysiologic association between migraine and the development of BPPV is not well understood. A study proposed that migraine is associated with the vasospasm of intracranial arteries, and repeated vasospasm can cause ischemic damage to the inner ear structure, eventually leading to the displacement of otoconia from the macular membrane (79). Moreover, head trauma has been increasingly linked to the onset of migraine (80), and may also act as a cofactor triggering the development of BPPV.

#### Inner ear diseases

BPPV can be caused by various inner ear diseases, including Meniere’s disease, sudden SNHL, labyrinthitis and vestibular neuritis. Consistent with our findings, a meta-analysis reported a significant correlation between Meniere’s disease and BPPV, with a pooled average frequency of 14% (95% CI 9–18%) (81). The relationship between Meniere’s disease and the occurrence of BPPV could be explained by secondary damage to the hydrops in the utricle and subsequent otoconia detachment (82). This review confirmed the previously reported relationship between neurolabyrinthitis and related conditions and BPPV occurrence (83). Mandalà et al. (84) observed that approximately 10% of vestibular neuritis patients developed BPPV, and its occurrence was associated with a lower age at onset.

Following our assessment of the included studies, we can assume that the relationship between SNHL and BPPV found by other researchers may be controversial. A study by Hong and Yeo suggested that BPPV does not commonly accompany SNHL, as only 5.4% of their sample had BPPV secondary to SNHL (85). However, in a review, Yetiser (86) reported a higher incidence of BPPV post-idiopathic SNHL, with onset ranging from days to several years after the occurrence of hearing loss. This variability in BPPV onset may account for the mixed findings in studies examining this correlation, as outcomes likely depend on the duration and follow-up periods of the research. Further studies are needed to better understand and clarify the relationship between SNHL and the subsequent development of BPPV.

#### Psychiatric comorbidities and insomnia

A systematic review reported an association between anxiety and BPPV but no significant association between depression and BPPV (87), aligning with our findings. The psychogenic hypothesis proposes that pre-existing psychological distress and secondary hyperventilation can result in vertigo or dizziness (88). This review suggests that the presence of obsessive-compulsive disorder (OCD) may increase the risk of BPPV occurrence. Although this association is not well studied, a single cohort study showed that 46% of patients with vertigo had OCD (89). However, as their findings were based on an uncontrolled study, the independent or causal association remains unclear and needs further investigation.

Observational studies have indicated a connection between poor sleep quality and an increased risk of BPPV, with 17% of BPPV patients exhibited insomnia (90, 91). These reports align with our finding that insomnia was associated with BPPV occurrence. Sleep deprivation is associated with increased vestibulo-ocular reflex asymmetry, neuroendocrine dysfunction, and inflammation of vestibular neurons, which are suggested to play a crucial role in the occurrence of BPPV (92, 93). Moreover, anxiety and depression are increasingly reported to lead to insomnia and sleep disorders and potentially act as cofactors triggering the development of BPPV (94, 95).

#### Cardiovascular and endocrine comorbidities

Several studies showed an association between BPPV occurrence and systemic diseases, including hyperlipidaemia, dyslipidaemia, hypertension, diabetes mellitus and stroke (6, 96, 97). However, recent meta-analyses indicate these are not risk factors for BPPV (98). Theoretically, hypertension, stroke and hyperlipidaemia can lead to inadequate blood supply and vascular damage to the inner ear, disrupting normal functioning and stability of the otoliths, potentially leading to the development of BPPV (99). A study of the temporal bone from patients with diabetes revealed a high prevalence of otoconia debris migration from the utricle in this population compared to healthy individuals (100). The literature suggests that this could be attributed to vasculopathy, neuropathy or reduced recovery from slight injury, trauma or infection induced by diabetes, which could increase the vulnerability of the vestibular system (101). Each of these factors individually or combined could affect inner ear function and potentially lead to BPPV. These results are consistent with the findings of our review, but contradict the results of a study by Chen and colleagues (98). The contrasting findings with Chen and colleagues study may be attributed to the influence of factors such as age, sample size, number and design of included studies and definition of BPPV, affecting the true association between BPPV and these factors.

An observational study showed that anaemia is not a risk factor for BPPV when comparing BPPV patients with a healthy group (*p* > 0.05) (102), although our review suggested an association between the two. To the best of our knowledge, this association has only been reported in a single study and thus awaits further confirmation. A case–control study reported a higher hypothyroidism rate in the BPPV group than in controls (21% vs. 0.02%, *p* < 0.001) (103), consistent with our findings. However, a recent study found no causal relationship between hypothyroidism and BPPV (104). The assessment and analysis measures, along with the inclusion/exclusion criteria for this study, may have influenced the observation. Lima et al. in their meta-analysis, reported controversial findings on the association between hypothyroidism and BPPV development but noted an association between BPPV with other thyroid diseases (105). Their statement contrasts with our findings of an association between hypothyroidism and BPPV development but no association of BPPV with thyroid disease. Based on these mixed results, the relationship between these factors and BPPV occurrence is controversial, and further studies are required to clarify this link.

Ischemic and coronary heart disease and hypotension are reported to cause inner ear vascular damage, potentially leading to BPPV (99, 106). Studies included in the present review showed that the evidence regarding this association is mixed. However, a nationwide cross-sectional study involving 1,003 participants demonstrated that coronary heart disease was not associated with BPPV after controlling for age and gender, with possible selection bias from self-reported data (6). The association between BPPV and cardiovascular conditions, such as hypertension and orthostatic hypotension in Parkinson’s disease patients, suggests that vascular instability can influence vestibular health (107). Moreover, Pezzoli et al. (108) found no significant association between orthostatic hypotension and residual dizziness post-BPPV recovery, although the prevalence of residual dizziness was higher (34%) than in the general population, indicating a potential role. No direct link between hypotension and BPPV has been confirmed, further research is required.

#### Cervical spondylosis

The present review suggests that cervical spondylosis increases the risk of developing BPPV. In line with our findings, observational studies have shown that a history of cervical spondylosis is a risk factor for BPPV, with significant differences between BPPV groups and controls (102). The degenerative changes in the cervical spine can compress the vertebral artery and cervical sympathetic nerve, causing ischemia in the vertebral-basilar system. Reduced blood flow to the vestibular system may cause otoliths to dislodge, triggering BPPV (102, 109).

#### Body mass index (BMI)

The evidence on the association between BMI and BPPV is mixed in this review. Studies showed no statistically significant association between BMI and BPPV compared to healthy groups (102, 110). This suggests that BMI may not directly trigger the development of BPPV. Higher BMI is often associated with comorbidities like diabetes and hypertension (111), which may act as cofactors to trigger the development of BPPV. Further research is needed to confirm or refute the direct association between BMI and BPPV.

#### Neuropathy and kidney disease

Literature shows that peripheral neuropathy often involves balance disorders and vertigo, possibly due to vestibular nerve degeneration, leading to BPPV (112). Our findings suggest an association between peripheral neuropathy and BPPV occurrence. However further studies are needed. This review also presents evidence indicating no association between kidney disease and BPPV incidence. To our knowledge, studies suggesting that kidney disease is directly associated with BPPV are limited; however, shared risk factors such as diabetes, cardiovascular disease and vitamin D deficiency suggest a potential indirect association (6, 113–115). Further research is needed to confirm the direct association between kidney disease and BPPV.

### Initial CRP outcomes

#### Head trauma, restricted neck mobility and idiopathic BPPV

An observational study on 104 patients found that the poorest success rate after one CRP session was associated with a history of labyrinthitis/neuronitis (57.1%), a history of trauma (35.7%), endolymphatic hydrops (30%) and idiopathic BPPV (20%) (116). The highest number of sessions was associated with labyrinthitis/neuronitis, followed by endolymphatic hydrops, trauma and idiopathic BPPV. Consistently, our review showed that idiopathic BPPV and a history of trauma were predictors of a poor prognosis after one CRP and that patients with a history of trauma required more CRP sessions to treat BPPV than patients with idiopathic BPPV.

The pathogenesis of posttraumatic BPPV may involve utricular microscopic haemorrhages from shear force, which enhances the formation of otoconial debris in the canal (117). Therefore, the poor CRP prognosis could be attributed to otoconial detachment and/or the presence of otoconial debris, leading to an increased number of treatment sessions (117, 118). Moreover, traumatic events may simultaneously cause neck pain and restricted mobility (119). Although patients with neck pain can benefit from the CRP, the efficacy of the initial treatment remains uncertain as these manoeuvres require movement of the head and neck (120). Korres et al. (121) found restricted neck mobility reduced initial CRP efficacy and increased the need for several sessions, highlighting that BPPV aetiology is an important prognostic factor for treatment outcomes. This is in line with our finding that neck pain and reduced cervical mobility were associated with poor CRP outcomes.

Controversially, Yoon et al. reported that the underlying aetiology does not affect CRP efficacy and the number of sessions required for BPPV treatment (122). In their study of 1900 patients, idiopathic BPPV required more sessions for symptom resolution than posttraumatic BPPV. However, they only investigated BPPV secondary to vestibular neuritis, sudden SNHL, and trauma, excluding comorbidities, such as cardiovascular diseases, limiting the generalisability of their findings. The poor outcomes in idiopathic BPPV could be attributed to several factors. First, detached otoconia may reverse direction if the manoeuvre is improperly performed. Second, an obstruction may exist within the membranous duct. Third, cupulolithiasis may underlie some BPPV cases resistant to CRP treatment, and finally, the location of the affected canal may contribute to CRP treatment failure (123, 124). The findings of our review suggest that idiopathic BPPV may be a predictor of a poorer CRP prognosis and an increased number of treatment sessions compared to BPPV associated with other comorbidities. However, the quality of studies included varied from poor-to-good, warranting cautious interpretation of these results. Furthermore, some studies on idiopathic BPPV did not fully account for the patient’s medical characteristics, raising the possibility of unreported comorbidities that could influence the treatment outcome. Different findings regarding prognosis after one CRP treatment could be attributable to variations in the treatment method/modifications, follow-up protocols or the number of enrolled patients. Further high-quality studies are needed in this area to confirm the true association between idiopathic BPPV and the efficacy of initial CRP treatment and whether idiopathic BPPV predicts poorer CRP outcomes compared to other comorbidities.

#### Cardiovascular and endocrine comorbidities

This review suggests that hypertension, coronary heart disease, hyperlipidaemia, dyslipidaemia, stroke, diabetes mellitus and hypothyroidism are risk factors for poor first CRP outcomes. Our findings agree with the work of Zhou et al., who observed that hypertension, diabetes and heart disease as factors influencing CRP prognosis (125). Similarly, Korkmaz and Korkmaz (126) reported that patients with hypertension required a higher number of treatment visits compared to patients without hypertension (*p* < 0.05) and reported no association with diabetes mellitus. In their study population, the prevalence of hypertension was reported as 26.1%, while diabetes has a prevalence of 11.1%. This discrepancy may have influenced their conclusions. Faralli et al. (127) in their study of 536 patients, found vascular comorbidities including hypertension, hyperlipidaemia, dyslipidaemia, ischemic heart disease and stroke increased the need for more than one CRP session, with those having multiple vascular comorbidities requiring significantly higher number of treatment sessions. On the contrary, Babac et al. (10) found no statistical association between CRP efficacy and hypertension, heart disease, hyperlipoproteinemia, cerebrovascular disease, diabetes mellitus and thyroid dysfunction (*p* > 0.05). The relationship between these factors and CRP efficacy is relatively controversial due to these mixed results. Different findings regarding the prognosis of CRP treatment could be attributable to variations in the treatment method, the definition of success, follow-up protocols, or the number of enrolled patients. Further studies are required for clarification.

#### Osteoporosis and serum vitamin D level

A cohort study of 400 patients, identified age over 50 years (*p* = 0.014), osteoporosis (*p* = 0.038) and head trauma (*p* = 0.000) as factors negatively affecting CRP outcome (10). This aligns with our findings demonstrating that osteoporosis is a predictor of poor first CRP outcomes. The present review demonstrates an association between low vitamin D levels and poor CRP outcomes, consistent with previous studies indicating that BPPV patients with vitamin D deficiency experience lower success rates with the initial CRP and have high recurrence rates (128, 129). Moreover, patients with vitamin D deficiency required more CRP sessions for successful treatment compared to those with normal serum vitamin D levels (*p* = 0.037) (130). Jeong et al. (71) proposed that calcium metabolism and vitamin D affect the homeostasis of otoconia metabolism, which regulates the formation and absorption of otoconia, potentially leading to otoconial detachment. Elevated concentrations of free calcium in the endolymph reduce the dilution capacity of the detached otoconia (131). Additionally, morphologic changes in otoconia (decreased density and increased size) can cause poor attachment to the utricular macula (71). This could potentially impact treatment efficacy and cause recurrent BPPV symptoms. Further clinical research is necessary to better understand this association.

#### SNHL and migraine

Consistent with our findings, previous studies indicate that CRP has a poor prognosis in patients with SNHL, often requiring multiple sessions for full recovery from BPPV symptoms (132–134). Lee and Ban in a review of 38 patients with sudden SNHL and BPPV, suggested that the co-occurrence of these conditions represents definite vestibular damage and is closely associated with a poor prognosis (135). In contrast, Domínguez-Durán et al. (136) demonstrated that inner ear diseases, including SNHL, did not affect initial CRP outcome. This discrepancy could be attributed to the study design, patient population, involved canal or type of CRP manoeuvres used and highlights the need for further research to clarify the impact of SNHL on CRP outcomes and determine the conditions under which SNHL may or may not affect treatment outcomes.

Interesting data also emerged concerning the impact of migraine on CRP outcomes. Domínguez-Durán et al. (136) found no significant differences in the rate of loss of nystagmus during initial CRP in BPPV patients with and without migraine. Other studies also reported no differences in recovery rates or the number of CRPs necessary for symptom resolution (127, 137). In the present review, we identified a single study by Gupta and Solanki suggesting that migraine serves as a predictor of poor CRP outcomes (29). This finding stands in contrast to the conclusions drawn by other researchers. It is important to note that this association was observed in a single study, based on patient’s baseline health characteristic at the time of initial CRP treatment, and was not subjected to statistical analysis. Further studies are required to clarify this link.

## Limitations

This review has some limitations. First, its scope was limited by the narrow time window of 5 years for studies in English language. Second, the results were very mixed, and there was a large degree of heterogeneity between studies, including variability in the definition and diagnosis of BPPV, involved semicircular canal, type of CRP, treatment modifications, and patient age. Additionally, some studies presented comorbid conditions solely as background health characteristics without statistical examination, limiting our ability to assess their impact on BPPV accurately. Finally, some studies on idiopathic BPPV did not account for the medical characteristics of included patients, unreported comorbidities could potentially influence treatment outcomes. Large-scale studies of these risk factors are needed to confirm the reliability of these results.

## Conclusion

This systematic review provides a comprehensive listing of recent studies into possible risk factors and comorbidities outlined in the literature that are associated with BPPV occurrence and poor outcome of the initial CRP. Some risk factors were identified for BPPV occurrence that are confirmed in more than one good quality study, including head trauma, vascular risk factors including hypertension and diabetes mellitus, stroke; vitamin D metabolism, osteoporosis and hypothyroidism, although there was some variability documented across multiple studies. Some factors were identified that have weaker or less consistent support from single, smaller or less robust studies including insomnia, peripheral neuropathy, neck disease, raised body mass index and some psychological disorders, with only negative results for renal disease and depression. Similarly, factors affecting outcome of initial CRP in more than one good quality study include head injury, idiopathic cases and vitamin D levels, and cardiovascular conditions. Consequently, the co-occurrence of multiple comorbidity could further increase the risk of BPPV and necessitate multiple CRP session for effective management. We recommend future studies on risk factors for BPPV use internationally agreed diagnostic criteria to facilitate meta analysis, and ensure study design minimises risk of bias; we also recommend that comorbidity, especially all the factors discussed in this review is recorded in the study population descriptors in any future trials of BPPV CRP outcomes given the potential that they may have to influence outcomes. Clinicians may also wish to consider paying particular attention to identified risk factors in this review when assessing patients with BPPV, especially those which are potentially modifiable. Future research could also aim to investigate candidate risk factors and comorbid conditions across BPPV subtypes and explore how these factors influence the efficacy of different treatment maneuvers.
