# Supplementary material for: Benign paroxysmal positional vertigo a systematic review of the effects of comorbidities
Source: Front Neurol. 2025 May 23;16:1595693. doi: 10.3389/fneur.2025.1595693 (PMC12141001; doi:10.3389/fneur.2025.1595693)
Supplement: Supplementary file 1 [file Table_1.docx]

**Supplemental Table 1:** Systematic review search and results

**Supplemental Table 2:** Assessment of bias in the cohort studies using the Newcastle–Ottawa Scale.

**Supplemental Table 3:** Assessment of bias in the case-control studies using the Newcastle–Ottawa Scale.

**Supplemental Table 4:** Assessment of bias in the cross-sectional studies using the Newcastle–Ottawa Scale.

**Supplemental Table 5:** Assessment of bias in the randomised controlled trials using the Cochrane risk-of-bias tool for randomised trials.

**Supplemental Table 1:** Systematic review search and results

| **Search** | **Search terms** | **Database** | **Results** | **Filters** |
| --- | --- | --- | --- | --- |
| 1 | (‘occurrence’ OR ‘incidence’) AND (‘risk factor’ OR ‘comorbidity’ OR ‘hypertension’ OR ‘diabetes’ OR ‘vitamin D’ OR ‘25-hydroxyvitamin D’ OR ‘ergocalciferol’ OR ‘cholecalciferol’ OR ‘migraine’ OR ‘osteoporosis’ OR ‘osteopenia’ OR ‘hyperlipidemia’ OR ‘cholesterol level’ OR ‘stroke’ OR ‘trauma’ OR ‘vascular’ OR ‘osteoarthritis’ OR ‘arthritis’ OR ‘idiopathic’ OR ‘anxiety’ OR ‘psychological’ OR ‘depression’ OR ‘psychiatric’ OR ‘autoimmune diseases’ OR “Menière’s disease” OR ‘hydrops’ OR ‘labyrinth diseases’ OR ‘inner ear disease’ OR ‘headache’) AND (‘bppv’ OR ‘benign paroxysmal positional vertigo’) | CINAHL | 18 | Years 2019–2024, English language |
| 2 | (‘risk factor’ OR ‘comorbidity’ OR ‘hypertension’ OR ‘diabetes’ OR ‘vitamin D’ OR ‘25-hydroxyvitamin D’ OR ‘ergocalciferol’ OR ‘cholecalciferol’ OR ‘migraine’ OR ‘osteoporosis’ OR ‘osteopenia’ OR ‘hyperlipidemia’ OR ‘cholesterol level’ OR ‘stroke’ OR ‘trauma’ OR ‘vascular’ OR ‘osteoarthritis’ OR ‘arthritis’ OR ‘back pain’ OR ‘neck pain’ OR ‘cervical’ OR ‘cervical spine injury’ OR ‘idiopathic’ OR ‘cervical extension’ OR ‘labyrinth diseases’ OR ‘anxiety’ OR ‘psychological’ OR ‘depression’ OR ‘psychiatric’ OR ‘autoimmune diseases’ OR “Menière’s disease” OR ‘hydrops’ OR ‘inner ear disease’ OR ‘headache’) AND (‘BPPV’ OR ‘benign paroxysmal positional vertigo’) AND (‘manoeuvre’ OR ‘maneuver’ OR ‘CRP’ OR ‘CRM’ OR ‘canalith repositioning’ OR ‘treatment’ OR ‘Epley’ OR ‘Semont’ OR ‘Brandt Daroff’ OR ‘Gans’ OR ‘barbecue’ OR ‘Gufoni’) AND (‘efficacy’ OR ‘outcome’ OR ‘success’) | CINAHL | 35 | Years 2019–2024, English language |
| 3 | (‘occurrence’ OR ‘incidence’) AND (‘risk factor’ OR ‘comorbidity’ OR ‘hypertension’ OR ‘diabetes’ OR ‘vitamin D’ OR ‘25-hydroxyvitamin D’ OR ‘ergocalciferol’ OR ‘cholecalciferol’ OR ‘migraine’ OR ‘osteoporosis’ OR ‘osteopenia’ OR ‘hyperlipidemia’ OR ‘cholesterol level’ OR ‘stroke’ OR ‘trauma’ OR ‘vascular’ OR ‘osteoarthritis’ OR ‘arthritis’ OR ‘idiopathic’ OR ‘anxiety’ OR ‘psychological’ OR ‘depression’ OR ‘psychiatric’ OR ‘autoimmune diseases’ OR “Menière’s disease” OR ‘hydrops’ OR ‘labyrinth diseases’ OR ‘inner ear disease’ OR ‘headache’) AND (‘bppv’ OR ‘benign paroxysmal positional vertigo’) | MEDLINE | 83 | Years 2019–2024, English language |
| 4 | (‘risk factor’ OR ‘comorbidity’ OR ‘hypertension’ OR ‘diabetes’ OR ‘vitamin D’ OR ‘25-hydroxyvitamin D’ OR ‘ergocalciferol’ OR ‘cholecalciferol’ OR ‘migraine’ OR ‘osteoporosis’ OR ‘osteopenia’ OR ‘hyperlipidemia’ OR ‘cholesterol level’ OR ‘stroke’ OR ‘trauma’ OR ‘vascular’ OR ‘osteoarthritis’ OR ‘arthritis’ OR ‘back pain’ OR ‘neck pain’ OR ‘cervical’ OR ‘cervical spine injury’ OR ‘idiopathic’ OR ‘cervical extension’ OR ‘labyrinth diseases’ OR ‘anxiety’ OR ‘psychological’ OR ‘depression’ OR ‘psychiatric’ OR ‘autoimmune diseases’ OR “Menière’s disease” OR ‘hydrops’ OR ‘inner ear disease’ OR ‘headache’) AND (‘BPPV’ OR ‘benign paroxysmal positional vertigo’) AND (‘manoeuvre’ OR ‘maneuver’ OR ‘CRP’ OR ‘CRM’ OR ‘canalith repositioning’ OR ‘treatment’ OR ‘Epley’ OR ‘Semont’ OR ‘Brandt Daroff’ OR ‘Gans’ OR ‘barbecue’ OR ‘Gufoni’) AND (‘efficacy’ OR ‘outcome’ OR ‘success’) | MEDLINE | 49 | Years 2019–2024, English language |
| 5 | (‘occurrence’ OR ‘incidence’) AND (‘risk factor’ OR ‘comorbidity’ OR ‘hypertension’ OR ‘diabetes’ OR ‘vitamin D’ OR ‘25-hydroxyvitamin D’ OR ‘ergocalciferol’ OR ‘cholecalciferol’ OR ‘migraine’ OR ‘osteoporosis’ OR ‘osteopenia’ OR ‘hyperlipidemia’ OR ‘cholesterol level’ OR ‘stroke’ OR ‘trauma’ OR ‘vascular’ OR ‘osteoarthritis’ OR ‘arthritis’ OR ‘idiopathic’ OR ‘anxiety’ OR ‘psychological’ OR ‘depression’ OR ‘psychiatric’ OR ‘autoimmune diseases’ OR “Menière’s disease” OR ‘hydrops’ OR ‘labyrinth diseases’ OR ‘inner ear disease’ OR ‘headache’) AND (‘bppv’ OR ‘benign paroxysmal positional vertigo’) | Web of Science | 108 | Years 2019–2024, English language |
| 6 | (‘risk factor’ OR ‘comorbidity’ OR ‘hypertension’ OR ‘diabetes’ OR ‘vitamin D’ OR ‘25-hydroxyvitamin D’ OR ‘ergocalciferol’ OR ‘cholecalciferol’ OR ‘migraine’ OR ‘osteoporosis’ OR ‘osteopenia’ OR ‘hyperlipidemia’ OR ‘cholesterol level’ OR ‘stroke’ OR ‘trauma’ OR ‘vascular’ OR ‘osteoarthritis’ OR ‘arthritis’ OR ‘back pain’ OR ‘neck pain’ OR ‘cervical’ OR ‘cervical spine injury’ OR ‘idiopathic’ OR ‘cervical extension’ OR ‘labyrinth diseases’ OR ‘anxiety’ OR ‘psychological’ OR ‘depression’ OR ‘psychiatric’ OR ‘autoimmune diseases’ OR “Menière’s disease” OR ‘hydrops’ OR ‘inner ear disease’ OR ‘headache’) AND (‘BPPV’ OR ‘benign paroxysmal positional vertigo’) AND (‘manoeuvre’ OR ‘maneuver’ OR ‘CRP’ OR ‘CRM’ OR ‘canalith repositioning’ OR ‘treatment’ OR ‘Epley’ OR ‘Semont’ OR ‘Brandt Daroff’ OR ‘Gans’ OR ‘barbecue’ OR ‘Gufoni’) AND (‘efficacy’ OR ‘outcome’ OR ‘success’) | Web of Science | 74 | Years 2019–2024, English language |
| 7 | (TITLE-ABS-KEY (‘occurrence’ OR ‘incidence’) AND TITLE-ABS-KEY (‘risk factor’ OR ‘comorbidity’ OR ‘hypertension’ OR ‘diabetes’ OR ‘vitamin D’ OR ‘25-hydroxyvitamin D’ OR ‘ergocalciferol’ OR ‘cholecalciferol’ OR ‘migraine’ OR ‘osteoporosis’ OR ‘osteopenia’ OR ‘hyperlipidemia’ OR ‘cholesterol level’ OR ‘stroke’ OR ‘trauma’ OR ‘vascular’ OR ‘osteoarthritis’ OR ‘arthritis’ OR ‘idiopathic’ OR ‘anxiety’ OR ‘psychological’ OR ‘depression’ OR ‘psychiatric’ OR ‘autoimmune diseases’ OR “Menière’s disease” OR ‘hydrops’ OR ‘labyrinth diseases’ OR ‘inner ear disease’ OR ‘headache’) AND TITLE-ABS-KEY (‘bppv’ OR ‘benign paroxysmal positional vertigo’)) | Scopus | 185 | Years >2018 and <2025, English language |
| 8 | (TITLE-ABS-KEY (‘risk factor’ OR ‘comorbidity’ OR ‘hypertension’ OR ‘diabetes’ OR ‘vitamin D’ OR ‘25-hydroxyvitamin D’ OR ‘ergocalciferol’ OR ‘cholecalciferol’ OR ‘migraine’ OR ‘osteoporosis’ OR ‘osteopenia’ OR ‘hyperlipidemia’ OR ‘cholesterol level’ OR ‘stroke’ OR ‘trauma’ OR ‘vascular’ OR ‘osteoarthritis’ OR ‘arthritis’ OR ‘back pain’ OR ‘neck pain’ OR ‘cervical’ OR ‘cervical spine injury’ OR ‘idiopathic’ OR ‘cervical extension’ OR ‘labyrinth diseases’ OR ‘anxiety’ OR ‘psychological’ OR ‘depression’ OR ‘psychiatric’ OR ‘autoimmune diseases’ OR “Menière’s disease” OR ‘hydrops’ OR ‘inner ear disease’ OR ‘headache’) AND TITLE-ABS-KEY (‘BPPV’ OR ‘benign paroxysmal positional vertigo’) AND TITLE-ABS-KEY (‘manoeuvre’ OR ‘maneuver’ OR ‘CRP’ OR ‘CRM’ OR ‘canalith repositioning’ OR ‘treatment’ OR ‘Epley’ OR ‘Semont’ OR ‘Brandt Daroff’ OR ‘Gans’ OR ‘barbecue’ OR ‘Gufoni’) AND TITLE-ABS-KEY (‘efficacy’ OR ‘outcome’ OR ‘success’)) | Scopus | 148 | Years >2018 and <2025, English language |
| 9 | (‘occurrence’ or ‘incidence’) AND (‘risk factor’ OR ‘comorbidity’ OR ‘hypertension’ OR ‘diabetes’ OR ‘vitamin D’ OR ‘25-hydroxyvitamin D’ OR ‘ergocalciferol’ OR ‘cholecalciferol’ OR ‘migraine’ OR ‘osteoporosis’ OR ‘osteopenia’ OR ‘hyperlipidemia’ OR ‘cholesterol level’ OR ‘stroke’ OR ‘trauma’ OR ‘vascular’ OR ‘osteoarthritis’ OR ‘arthritis’ OR ‘idiopathic’ OR ‘anxiety’ OR ‘psychological’ OR ‘depression’ OR ‘psychiatric’ OR ‘autoimmune diseases’ OR “Menière’s disease” OR ‘hydrops’ OR ‘labyrinth diseases’ OR ‘inner ear disease’ OR ‘headache’) AND (‘bppv’ OR ‘benign paroxysmal positional vertigo’) | PubMed | 239 | Years 2019–2024, English language |
| 10 | (‘risk factor’ OR ‘comorbidity’ OR ‘hypertension’ OR ‘diabetes’ OR ‘vitamin D’ OR ‘25-hydroxyvitamin D’ OR ‘ergocalciferol’ OR ‘cholecalciferol’ OR ‘migraine’ OR ‘osteoporosis’ OR ‘osteopenia’ OR ‘hyperlipidemia’ OR ‘cholesterol level’ OR ‘stroke’ OR ‘trauma’ OR ‘vascular’ OR ‘osteoarthritis’ OR ‘arthritis’ OR ‘back pain’ OR ‘neck pain’ OR ‘cervical’ OR ‘cervical spine injury’ OR ‘idiopathic’ OR ‘cervical extension’ OR ‘labyrinth diseases’ OR ‘anxiety’ OR ‘psychological’ OR ‘depression’ OR ‘psychiatric’ OR ‘autoimmune diseases’ OR “Menière’s disease” OR ‘hydrops’ OR ‘inner ear disease’ OR ‘headache’) AND (‘BPPV’ OR ‘benign paroxysmal positional vertigo’) AND (‘manoeuvre’ OR ‘maneuver’ OR ‘CRP’ OR ‘CRM’ OR ‘canalith repositioning’ OR ‘treatment’ OR ‘Epley’ OR ‘Semont’ OR ‘Brandt Daroff’ OR ‘Gans’ OR ‘barbecue’ OR ‘Gufoni’) AND (‘efficacy’ OR ‘outcome’ OR ‘success’) | PubMed | 173 | Years 2019–2024, English language |
| 11 | ((‘occurrence’ or ‘incidence’) AND (‘risk factor’ OR ‘comorbidity’ OR ‘hypertension’ OR ‘diabetes’ OR ‘vitamin D’ OR ‘25-hydroxyvitamin D’ OR ‘ergocalciferol’ OR ‘cholecalciferol’ OR ‘migraine’ OR ‘osteoporosis’ OR ‘osteopenia’ OR ‘hyperlipidemia’ OR ‘cholesterol level’ OR ‘stroke’ OR ‘trauma’ OR ‘vascular’ OR ‘osteoarthritis’ OR ‘arthritis’ OR ‘idiopathic’ OR ‘anxiety’ OR ‘psychological’ OR ‘depression’ OR ‘psychiatric’ OR ‘autoimmune diseases’ OR “Menière’s disease” OR ‘hydrops’ OR ‘labyrinth diseases’ OR ‘inner ear disease’ OR ‘headache’) AND (‘bppv’ OR ‘benign paroxysmal positional vertigo’)) | Embase | 143 | Years 2019–2024, English language |
| 12 | ((‘risk factor’ OR ‘comorbidity’ OR ‘hypertension’ OR ‘diabetes’ OR ‘vitamin D’ OR ‘25-hydroxyvitamin D’ OR ‘ergocalciferol’ OR ‘cholecalciferol’ OR ‘migraine’ OR ‘osteoporosis’ OR ‘osteopenia’ OR ‘hyperlipidemia’ OR ‘cholesterol level’ OR ‘stroke’ OR ‘trauma’ OR ‘vascular’ OR ‘osteoarthritis’ OR ‘arthritis’ OR ‘back pain’ OR ‘neck pain’ OR ‘cervical’ OR ‘cervical spine injury’ OR ‘idiopathic’ OR ‘cervical extension’ OR ‘labyrinth diseases’ OR ‘anxiety’ OR ‘psychological’ OR ‘depression’ OR ‘psychiatric’ OR ‘autoimmune diseases’ OR “Menière’s disease” OR ‘hydrops’ OR ‘inner ear disease’ OR ‘headache’) AND (‘BPPV’ or ‘benign paroxysmal positional vertigo’) AND (‘manoeuvre’ OR ‘maneuver’ OR ‘CRP’ OR ‘CRM’ OR ‘canalith repositioning’ OR ‘treatment’ OR ‘Epley’ OR ‘Semont’ OR ‘Brandt Daroff’ OR ‘Gans’ OR ‘barbecue’ OR ‘Gufoni’) AND (‘efficacy’ OR ‘outcome’ OR ‘success’)) | Embase | 128 | Years 2019–2024, English language |

# Supplemental Table 2 Assessment of bias in the cohort studies using the Newcastle–Ottawa Scale.

Green (*) symbols indicates a low risk of bias for the given criteria; yellow (_) symbols indicates that the study does not meet the criteria for a low risk of bias; (n/a) indicates that the criterion does not apply to the study (for example, retrospective studies do not have a follow-up period by design). Scores of 7-9 indicate low risk of bias (green); 4-6 unclear risk of bias; and ≤3 high risk of bias (red).

|  | **SELECTION** | | | | **COMPARABILITY** | **OUTCOME** | | | **QUALITY SCORE** |
| --- | --- | --- | --- | --- | --- | --- | --- | --- | --- |
|  | Representativeness of the exposed cohort | Selection of the non-exposed cohort | Ascertainment of exposure | Outcome of interest not present at the start of the study | Comparability of cohorts/confounders | Assessment of outcome | Sufficient follow-up | Adequacy of follow-up of cohorts |  |
| Kim, Pasquesi and Sharon, 2022 | * | * | _ | _ | * | * | * | n/a | 5 |
| Andersson et al., 2022 | * | * | * | * | * | * | * | * | 8 |
| Kim et al., 2024 | * | * | * | * | * | * | * | * | 8 |
| Álvarez-Morujo de Sande et al., 2019 | * | * | * | _ | * | * | * | * | 7 |
| Elmoursy and Abbas, 2021 | * | * | * | _ | ** | * | * | * | 8 |
| McCormick and Kolar, 2023 | * | * | * | _ | ** | * | * | * | 8 |
| Yehuda, Rachima and Katz-Leurer, 2024 | * | * | * | _ | ** | _ | * | * | 7 |
| Shu et al., 2023 | * | * | * | * | ** | * | * | * | 9 |
| Kong, Song and Shim, 2022 | * | * | * | _ | ** | * | * | * | 8 |
| El-Anwar et al., 2022 | * | * | * | _ | ** | * | * | * | 8 |
| Yang et al., 2019 | * | * | * | _ | * | * | * | * | 7 |
| Zhang et al., 2023 | * | * | * | _ | * | * | * | * | 7 |
| Gupta and Solanki, 2022 | * | * | * | _ | ** | * | * | * | 8 |
| Martens et al., 2019 | * | * | * | _ | * | _ | * | _ | 5 |
| Ghosh and Dorasala, 2023 | * | * | * | _ | * | * | * | * | 7 |
| Martellucci et al., 2022 | * | * | _ | * | * | _ | * | * | 6 |
| Nahm et al., 2019 | * | * | * | * | * | * | * | * | 8 |
| Fu et al., 2022 | * | * | * | * | ** | _ | * | * | 8 |
| Wu et al., 2023 | * | * | * | * | ** | _ | * | * | 8 |
| Lee et al., 2021 | * | * | * | * | ** | * | * | * | 9 |
| Maas et al., 2021 | * | * | * | * | * | * | * | * | 8 |
| De Hertogh et al., 2022 | * | * | * | * | _ | * | * | n/a | 6 |
| Zhao et al., 2021 | * | * | * | _ | * | * | * | * | 7 |
| Martellucci et al., 2019 | * | * | * | * | ** | * | * | * | 9 |
| Jiang et al., 2022 | * | * | * | * | ** | _ | * | * | 8 |

# Supplemental Table 3 Assessment of bias in the case-control studies using the Newcastle–Ottawa Scale.

Green (*) symbols indicates a low risk of bias for the given criteria; yellow (_) symbols indicates that the study does not meet the criteria for a low risk of bias. Scores of 7-9 indicate low risk of bias (green); 4-6 unclear risk of bias; and ≤3 high risk of bias (red).

|  | **SELECTION** | | | | **COMPARABILITY** | **EXPOSURE** | | | **QUALITY SCORE** |
| --- | --- | --- | --- | --- | --- | --- | --- | --- | --- |
|  | Adequate case definition | Representativeness of cases | Selection of controls | Definition of controls | Comparability of cases and controls based on the design or analysis | Ascertainment of exposure | Same method of ascertainment for cases and controls | Non-response rate |  |
| Ding et al., 2019 | * | _ | _ | * | ** | * | * | * | 7 |
| Bi et al., 2021 | * | * | * | * | ** | * | * | * | 9 |
| Cheng, Wang and Yu, 2021 | * | * | * | * | * | * | * | * | 8 |
| Ren et al., 2023 | * | _ | * | * | ** | * | * | * | 8 |
| Singh, Corser and Monsell, 2020 | * | * | * | * | ** | _ | * | * | 8 |
| Saruhan et al., 2021 | * | * | _ | * | * | * | * | * | 7 |

# Supplemental Table 4 Assessment of bias in the cross-sectional studies using the Newcastle–Ottawa Scale.

Green (*) symbols indicates a low risk of bias for the given criteria; yellow (_) symbols indicates that the study does not meet the criteria for a low risk of bias. Scores of 7-8 indicate low risk of bias (green); 4-6 unclear risk of bias; and ≤3 high risk of bias (red).

|  | **SELECTION** | | | | **COMPARABILITY** | **OUTCOME** | | **QUALITY** **SCORE** |
| --- | --- | --- | --- | --- | --- | --- | --- | --- |
|  | Representativeness of the sample | Sample size | Ascertainment of exposure | Non-respondents | Comparable subjects in different outcome groups based on the study design or analysis; controlled confounding factors | Assessment of outcomes | Reporting of results |  |
| Kher, 2023 | * | _ | * | * | _ | * | _ | 4 |
| Hyland, Hawke and Taylor, 2024 | * | * | _ | * | ** | * | * | 7 |
| Harrell et al., 2023 | * | _ | * | * | _ | * | * | 5 |
| Jafarzadeh, Pourbakht and Bahrami, 2022 | * | _ | * | * | _ | * | * | 5 |
| Jensen and Hougaard, 2022 | * | _ | * | * | _ | * | _ | 4 |
| Cobb et al., 2023 | * | _ | * | * | ** | * | * | 7 |

# Supplemental Table 5 Assessment of bias in the randomised controlled trials using the Cochrane risk-of-bias tool for randomised trials.

Green (-) symbols indicates a low risk of bias; red (+) symbols indicates a high risk of bias; yellow (?) symbols indicates an unclear risk of bias.

|  | Randomisation process | Deviations from the intended interventions | Missing outcome data | Measurement of the outcome | Selection of the reported result | **QUALITY SCORE** |
| --- | --- | --- | --- | --- | --- | --- |
| Lou et al., 2020 | - | - | - | ? | + | + |
| Lee et al., 2020 | - | - | - | - | - | - |
| Lee et al., 2022 | - | - | - | - | + | ? |
| Khaftari et al., 2021 | - | - | - | - | + | ? |
| Kjærsgaard, Petersen and Hougaard, 2023 | - | - | - | - | + | ? |
| Lee J et al., 2021 | - | - | - | - | + | ? |
| Chen et al., 2023 | - | - | - | - | + | ? |
| Imai et al., 2023 | - | - | - | - | + | ? |
| Nadagoud, Bhat and Pragathi, 2024 | - | - | - | - | + | ? |
| Han et al., 2024 | - | - | - | - | + | ? |
| Celis-Aguilar et al., 2022 | - | - | - | - | + | ? |
| Kong et al., 2020 | - | - | - | + | + | + |
| Schuricht and Hougaard, 2022 | - | - | - | - | + | ? |
